# Deciphering the Post-Operative Dynamics of Opportunistic Gut Microbiota in Colorectal Cancer Patients

**DOI:** 10.3390/microorganisms13122818

**Published:** 2025-12-11

**Authors:** Mutebi John Kenneth, Chuan-Yin Fang, Chin-Chia Wu, Ming-Chih Hsieh, Ming-Liang Lai, Bing-Mu Hsu

**Affiliations:** 1 Department of Earth and Environmental Sciences, National Chung Cheng University, 168 University Road, Minhsiung Township, Chiayi 62102, Taiwan; mjkenneth156@gmail.com; 2 Doctoral Program in Science, Technology, Environment and Mathematics, National Chung Cheng University, Chiayi 62102, Taiwan; 3 Division of Colon and Rectal Surgery, Ditmanson Medical Foundation Chia-Yi Christian Hospital, Chiayi 60002, Taiwan; 04969@cych.org.tw; 4 Division of Colorectal Surgery, Dalin Tzu Chi Hospital, Buddhist Tzu Chi Medical Foundation, Chiayi 62247, Taiwan; 5 College of Medicine, Tzu Chi University, Hualien 97004, Taiwan; 6 School of Post-Baccalaureate Chinese Medicine, Tzu Chi University, Hualien 97004, Taiwan; 7 Department of Radiology, Ditmanson Medical Foundation Chia-Yi Christian Hospital, Chiayi 60002, Taiwan; 8 Graduate Institute of Intellectual Property, National Taipei University of Technology, Taipei 10608, Taiwan

**Keywords:** colorectal cancer, gut microbiota, opportunistic bacteria, surgical resection, post-surgery dysbiosis

## Abstract

Recent studies indicate that opportunistic gut bacteria contribute to the recurrence and chemoresistance in colorectal cancer (CRC); however, their fate after surgical resection remains poorly understood. This study investigated the longitudinal changes in these bacteria and assessed their potential persistence following CRC surgery. Forty fecal samples were collected from ten CRC patients at four timepoints: (1) pre-surgery (S); (2) one week (S1); (3) one month (S2); and (4) three months (S3) post-surgery. Fifteen other fecal samples were collected from healthy individuals as our study controls. Microbial profiling was performed using 16S rRNA gene sequencing, and quantitative PCR was applied to assess the changes in three opportunistic bacteria associated with CRC-associated. Our study revealed that *Escherichia coli* was significantly enriched in pre-surgical samples (S), while *Enterococcus faecalis* was predominant in the samples collected one-week after surgery (S1). All the assessed species showed a gradual post-surgical decline in relative abundance, suggesting they do not persist after resection. Additionally, there was a significant increase in relative abundance of beneficial bacterial signatures, including *Akkermansia muciniphila*, *Bacteroides uniformis*, *Parabacteroides merdae*, and *Phascolarctobacterium faecium* post-surgery, which implies a potential dysbiosis restoration. Our findings suggest that surgical resection gradually reduces the burden of opportunistic gut microbiota, thus gradually lowering the risk of recurrence and chemoresistance. Additionally, it may facilitate the restoration of beneficial taxa. Future studies should include extended follow-up periods to validate our findings and their correlation with clinical outcomes.

## 1. Introduction

Colorectal cancer (CRC) is a major global health concern, ranking as the second most common cause of cancer-related deaths [1]. Its development is influenced by multiple factors, including sedentary lifestyle, heredity, diet, and gut microbiota composition [2]. Growing evidence suggests that Western dietary habits characterized by high consumption of refined sugars, processed meats, alcoholic beverages, and high-fat food contribute significantly to the risk of CRC development [3,4,5]. Such diet disrupts gut microbial balance, leading to dysbiosis and gut barrier dysfunction [6]. This eventually leads to low-grade systemic inflammation and encourages the overgrowth of opportunistic bacteria [7].

Several opportunistic gut bacteria such as *Polyketide synthase-positive* (*pks^+^*) *Escherichia coli*, *Fusobacterium nucleatum*, *Bacteroides fragilis*, and *Enterococcus faecalis*, have been strongly linked to CRC pathogenesis [8,9,10,11]. These bacteria are frequently enriched in CRC patient samples as compared to the healthy controls and may serve as diagnostic or prognostic biomarkers for CRC [12]. For example, *pks^+^ E. coli* are frequently harbored in the colonic mucosa of CRC patients compared to non-cancerous study controls [8,13,14]. They encode enzymes for colibactin, a genotoxin that indices DNA damages and mutational signatures found in CRC cells [15]. Similarly, *F. nucleatum* has been associated with poorer CRC survival and chemoresistance [16,17,18]. Other opportunistic bacteria such as *Streptococcus bovis*, *B. fragilis*, and *E. faecalis* have all been correlated with CRC development and patient survival [19,20,21]. Therefore, understanding the dynamics of these bacteria during and after CRC treatment could inform targeted therapies and improve patient outcomes.

Surgical resection remains the primary curative treatment, especially in early-stage CRC [22]. The surgical procedure involves preoperative bowel preparation including mechanical cleansing and oral antibiotics [23,24]. Recent studies have indicated that bowel preparation alters post-surgical microbiota in ways that may even affect wound healing and anastomotic integrity [25,26,27]. Previous studies have assessed the nature of post-surgery gut microbiota [28,29]; however, the fate of CRC-associated opportunistic bacteria remains poorly characterized.

Given that the tumor microenvironment ‘selectively fosters’ these opportunistic bacteria at the expense of beneficial ones [30,31], it is postulated that surgical resection might eliminate their ecological niche thus reducing their abundance in the patient gut [32]. However, if these pathogens persist, they could contribute to recurrence or chemoresistance, given their known contribution towards the disease pathogenesis [17,33,34]. To address this knowledge gap, we conducted a longitudinal assessment of gut microbiota in CRC patients, pre-surgery and at three post-surgical time points, i.e., one week, one month, and three months. Additionally, we analyze microbiota composition in non-cancerous family volunteers of these patients to establish comparative baselines. We employed 16S rRNA gene sequencing to characterize microbial community structure, while real-time quantitative PCR enabled us to quantitatively analyze the longitudinal changes in CRC-associated opportunistic bacterial species in the patients. Furthermore, functional and metabolic profiling of the microbiota was performed to identify enriched pathways supporting bacterial growth and host–microbe interactions in CRC patients compared to healthy controls. Our study provides a glimpse of the fate CRC-associated gut microbiota following curative resection.

## 2. Materials and Methods

### 2.1. Study Design and Fecal Sample Collection

This single-center, longitudinal pilot study was conducted at Chiayi Christian Hospital (Chiayi, Taiwan) between September 2023 and August 2024. CRC staging was determined using the American Joint Committee on Cancer (AJCC) 8th edition Tumor–Node–Metastasis (TNM) classification [35]. Patients diagnosed with CRC at TNM stages I–IV were eligible for inclusion. Their clinical and histopathological staging was determined through perioperative imaging, surgical records, and pathological assessments. The exclusion criteria included the presence of distant metastases, hereditary nonpolyposis CRC, other intestinal conditions (e.g., ischemic colitis, inflammatory bowel disease, or tuberculous colitis), and the use of antibiotics, probiotics, or other long-term medications (>3 consecutive days) within three months prior to enrollment. None of the patients received neoadjuvant chemotherapy, radiotherapy, or immunotherapy prior to enrollment into our study. The patients’ microsatellite stability (MSS/MSI status) was not assessed in this cohort.

All participants (or their legal guardians where applicable) provided written informed consent. Our study healthy controls consisted of volunteer family members of the enrolled patients. The study protocol was approved by the hospital’s ethics committee (IRB approval number: IRB2021088), and conducted in accordance with the Declaration of Helsinki [36].

Stool samples were collected from CRC patients at four time points: one week prior to initiation of any cancer treatment (baseline, S), and at one week (S1), one month (S2), and three months (S3) postoperatively, making a total of 40 patient fecal samples, with each patient contributing 4 samples overall. Baseline stool samples were also collected from the study healthy controls (*n* = 15). These four sampling time points were chosen to capture distinct phases of gut microbiome disturbance and recovery across the perioperative course, according to the advice from our collaborating surgeons. S served as the baseline prior to major interventions. Additionally, S1 was chosen to reflect the immediate postoperative disruption, coinciding with broad-spectrum prophylactic antibiotics, surgical trauma, and early stabilization. S2 was chosen to represent the initial reassembly phase, when antibiotics had largely cleared, diet normalized, and intestinal physiology began stabilizing. S3 was chosen to assess the medium-term adaptive equilibrium, indicating whether the microbiome returned toward baseline or established a new stable state. None of the patients experienced adverse postoperative complications or received adjuvant chemotherapy during the study period.

All baseline fecal samples were collected prior to colonoscopy and before polyethylene glycol consumption in both patients and healthy control to prevent the effects of bowel preparation to baseline gut microbial composition [37]. Samples were collected within one hour of morning defecation into single-use specimen collector pans and were emailed to our laboratory within one week (±3 days). They were stored at −80 °C until DNA extraction. None of our samples was subjected to freeze–thaw process before DNA extraction. Demographic and clinical data including age, sex, pathological subtype, and TNM stage were recorded at baseline for all patients.

### 2.2. Bacterial DNA Extraction from Fecal Samples

Bacterial DNA was extracted from 200 mg of each fecal sample using the QIAamp DNA Stool Mini Kit (QIAGEN, Hilden, Germany) following the manufacturer’s protocol, with modifications to enhance cell lysis and DNA yield. Specifically, a bead-beating step was incorporated based on a previously established protocol to improve the mechanical disruption of microbial cells [38]. Briefly, 250 µL of stool sample was transferred into a sterile 2 mL microcentrifuge tube, followed by the addition of 1.2 mL of ASL lysis buffer and 0.3 g of sterile 0.1 mm zirconia beads (BioSpec, Bartlesville, OK, USA). The mixture was vortexed for 2 min, heated at 95 °C for 15 min to enhance lysis, and subsequently homogenized using a Qiagen TissueLyser II.

Following homogenization, samples were treated with an InhibitEX Tablet to remove PCR inhibitors. A 350 µL aliquot of the resulting supernatant was then transferred to a fresh tube for automated DNA purification using the QIAcube system (QIAGEN). The concentration and purity of the extracted DNA were assessed using a NanoDrop 2000 spectrophotometer (Thermo Fisher Scientific, Wilmington, DE, USA), with absorbance readings measured at 230–280 nm to ensure suitability for downstream metagenomic applications. The quantified DNA was stored at −20 °C for 7 days before being packaged in dry ice and delivered to external vendors for sequencing.

### 2.3. Bacterial Detection by Polymerase Chain Reaction

Bacterial detection was performed using 16S rRNA gene amplification on a 2720 Thermal Cycler PCR machine (Applied Biosystems, Foster City, CA, USA), using a universal bacterial primer set: 27F (5′-AGRGTTYGATYMTGGCTCAG-3′) and 1492R (5′-RGYTACCTTGTTACGACTT-3′). The PCR conditions for this universal amplification included an initial denaturation at 95 °C for 3 min, followed by 35 cycles of denaturation at 94 °C for 30 s, annealing at 57 °C for 30 s, and extension at 72 °C for 30 s, with a final extension step at 72 °C for 10 min. Additionally, targeted PCR assays were conducted to identify CRC-associated bacterial species, using primers specific for *polyketide synthase-positive* (*pks⁺*) *E. coli* (*clb_B*), *B. fragilis* (*bft*), and *E. faecalis* (*16S rRNA*) as indicated in Table S1. The amplification conditions were as follows: initial denaturation at 95 °C for 3 min; 40 cycles of denaturation at 95 °C for 45 s, annealing at 55 °C for 30 s, and extension at 72 °C for 30 s; followed by a final extension at 72 °C for 8 min. PCR products of the correct sizes were identified on agarose gels to confirm the presence of the target bacterial species.

### 2.4. Quantitative Real-Time PCR Analysis of CRC-Associated Gut Microbiota

Quantitative real-time PCR (qPCR) was performed using the Applied Biosystems StepOnePlus™ Real-Time PCR System (Applied Biosystems, Foster City, CA, USA) to quantify three CRC-associated gut bacteria: *Polyketide synthase-positive* (*pks⁺*) *E. coli*, *B. fragilis*, and *E. faecalis*. The reactions were performed in a total volume of 20 µL using a 2× SYBR Green master mix (final 1×). Each reaction contained approximately 5 ng of template DNA (2 µL), forward and reverse primers at 0.5 µM final concentration each (from 10 µM stocks; 1 µL per primer), and nuclease-free water to volume. Typical per-reaction volumes were: 10 µL SYBR master mix (2×), 1 µL forward primer, 1 µL reverse primer, 2 µL template DNA (~5 ng), and 6 µL nuclease-free water. Reactions were run in duplicate for each sample, and mean cycle threshold (Ct) values were calculated. Thermal cycling conditions and oligonucleotide primers were adapted from previous studies, as indicated in Table S1. Specifically, *pks⁺ E. coli* was detected through amplification of the *clbB* gene (encoding colibactin B), while *B. fragilis* (encoding *B. fragilis* toxin) and *E. faecalis* were detected by targeting the *bft* and *efa* genes, respectively.

Positive controls for the *clbB*, *bft,* and *efa* genes were generated by T&A cloning of PCR-amplified gene fragments using the T&A™ Cloning Vector Kit (Yeastern Biotech Co. Ltd., Taipei, Taiwan), following the manufacturer’s instructions (https://yeastern.com/?page_id=1384, accessed on 5 October 2024). Quantification of total bacterial DNA was performed via 16S rRNA gene amplification and used for normalization of target gene expression levels. qPCR data were analyzed using StepOne™ Software v2.1 (https://www.thermofisher.com/tw/zt/home/technical-resources/software-downloads, accessed on 12 October 2024). The bacterial biomass per fecal gram was estimated based on the qPCR results of the 16S rRNA universal primer. To calculate the relative abundance of our target bacteria, we used the following formula: relative abundance = Cs/Bs × 100%, where Cs represents the copies of the specie and Bs represents the bacterial biomass [39].

### 2.5. Metagenomic Sequencing and Raw Data Processing

Fecal bacterial communities were characterized by full-length 16S rRNA gene amplification and sequencing. PCR amplicons of the 16S rRNA (20 μL per sample) were sequenced using the single-end PacBio platform [40], which was performed at the Genomics BioSci & Tech Co., New Taipei City, Taiwan. The obtained raw single-end FASTQ files were processed using the DADA2 plugin in QIIME2 following a previous study, which included quality trimming, merging, and chimera removal to generate high-quality sequences [41]. These sequences were then clustered into amplicon sequence variants (ASVs) at a 98% similarity threshold and assigned taxonomies by aligning representative sequences to the NCBI RefSeq database using the RefSeq classifier.

Taxonomic assignments were collapsed to the species level (levels 1–7) using the qiime taxa collapse command, enabling compositional and diversity analysis of the gut microbiota across different sampling time points. Taxonomic composition and diversity metrics were visualized using QIIME2 View (https://view.qiime2.org/, accessed on 15 December 2024), while Microsoft Excel 2010 was used to generate bar plots depicting the relative abundance of bacterial taxa across samples.

### 2.6. Statistical Analysis

Beta diversity was calculated by Bray–Curtis distances and was visualized through ordination using principal coordinate analysis (PCoA). Additionally, alpha diversity was assessed using Chao1, to account for species richness and evenness. Differences in diversity metrics between CRC patients (at each sampling time point) and controls were evaluated using the Wilcoxon rank-sum test. Paired analyses were employed for intra-patient comparisons across different time points to account for repeated measures. In this study, taxonomic comparisons were restricted to bacterial taxa with an average relative abundance greater than 0.5%, and statistical significance with *p*-value < 0.05.

Additionally, the quantitative real-time PCR results were analyzed to identify the altered abundance of our targeted opportunistic species across sampling time points. Kruskal–Wallis test was used where appropriate. Pearson correlation and Analysis of variance (ANOVA) were conducted using IBM SPSS Statistics v24, while unpaired two-tailed t-tests were performed in Microsoft Excel 2010. All other statistical analyses were carried out in R (v4.5.1). No Bonferroni correction or other false-discovery rate adjustment was applied, as the number of comparisons was limited and the analyses were intended to be exploratory.

## 3. Results

### 3.1. Study Cohort

This study included 25 participants, as summarized in Table S2 below. Among them, 10 were CRC patients (5 males and 5 females), and 15 healthy family members of the enrolled patients, serving as controls. Of these 10, 2 were diagnosed with Stage I, 4 with Stage II, and 4 with Stage III disease. Colonoscopy examination revealed that all CRC tumors were located in the rectum. The median hospital stay for CRC patients was 4 days (range: 2–14 days). The healthy control group had a mean age of 51.3 years (interquartile range [IQR]: 26.0) and a mean body mass index (BMI) of 23.6 (IQR: 6.0). In contrast, CRC patients had a mean age of 63.0 years (IQR: 8.5) and a mean BMI of 23.8 (IQR: 3.0).

### 3.2. Microbial Community Composition and Diversity

This study employed full-length 16S rRNA gene sequencing using the PacBio platform to characterize taxonomic profiles and longitudinal changes in gut microbiota of CRC patients. Analyzing these taxonomic profiles provided species-level insights into gut bacterial diversity of the patients at different time points, i.e., baseline (S), one week (S1), one month (S2), and three months post-surgery (S3).

**Figure 1:**
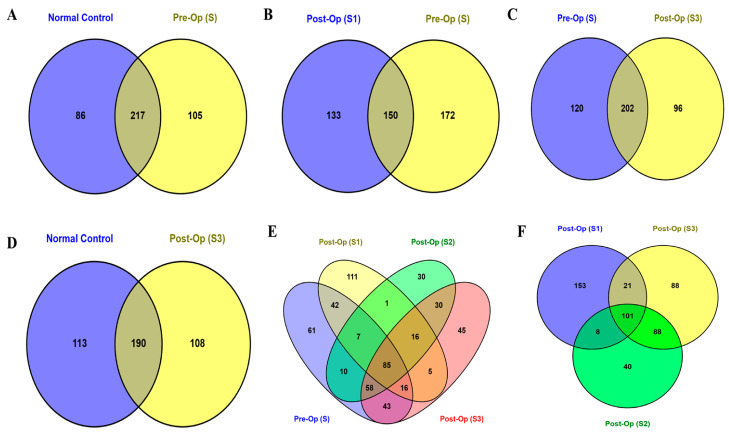
Comparative Venn diagrams illustrating bacterial taxa overlaps between healthy controls and CRC patients at different sampling timepoints. The Venn diagrams indicate unique and common bacterial species between (**A**) healthy controls (Normal control) and CRC patients at baseline (Pre-Op (S)); (**B**) patient samples at baseline (S) and one week after surgery (Post-Op (S1)); (**C**) patient samples (S) and 3 months after surgery (Post-Op (S3)); (**D**) patient samples 3 months after surgery (S3) and healthy controls; (**E**) all patient samples and (**F**) only post-surgery patient samples.

A total of 611 bacterial species were identified across all sample groups. Of these, 408 species were detected in both healthy controls and S samples. The latter exhibited a higher proportion of unique species (25.7%) compared to healthy controls (21.1%), although a substantial proportion of species (53.2%) were shared between the two groups (Figure 1A).

Postoperative longitudinal analysis revealed a decline in species richness over time. For example, S samples contained more unique species (37.8%) than S1 samples (29.2%) (Figure 1B). Similarly, the number of unique species at S3 (*n* = 96) was lower than in S samples (*n* = 120), and slightly below that of healthy controls (Figure 1C,D). Across all patient sampling time points, 85 unique bacterial species were identified, with the highest count observed in S1 samples (*n* = 111), followed by S (*n* = 61), S3 (*n* = 45), and S2 (*n* = 30) (Figure 1 E). However, the total number of species per group showed a different trend: healthy controls (*n* = 322), S samples (*n* = 283), S1 samples (*n* = 237), S2 samples (*n* = 298), and S3 samples (*n* = 303). These postoperative samples shared 101 unique bacterial species in total, with 153, 40, and 88 species uniquely identified at S1, S2, and S3, respectively (Figure 1 F).

### 3.3. The Shift in the Gut Microbial Diversity and Richness in Response to Colorectal Resection

**Figure 2:**
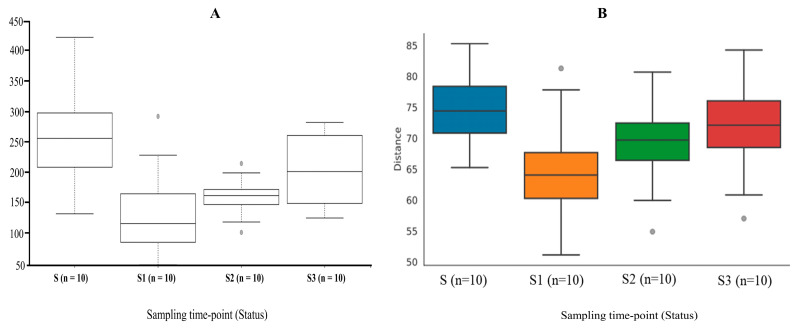
Bacterial alpha and beta diversities: (**A**) Box-plot representing alpha diversity index of Chao1 (*p*  =  0.005) among patient sampling groups. (**B**) Box-plot representing beta diversity index of Bray–Curtis distance matrix (*p*  =  0.003) among patient sampling groups.

Gut bacterial diversity and richness following surgical resection were assessed using the Chao1 index as a measure of alpha diversity. A significant reduction in diversity was observed in S1 samples compared to S, S2, and S3 samples (Figure 2A). However, no statistically significant differences were found between patient groups and healthy controls (*p* > 0.05). Kruskal–Wallis pairwise analysis revealed significant differences between S samples and both S1 (*p* = 0.004) and S2 (*p* = 0.002), and between S1 (*p* = 0.01) and S3 (*p* = 0.009) samples. Beta diversity analysis based on the Bray–Curtis dissimilarity index showed distinct clustering among CRC patient samples (PERMANOVA, pseudo -F). Consistent with alpha diversity findings, S1 samples exhibited a markedly distinct community structure (*p* = 0.024) relative to other patient groups (Figure 2B).

**Figure 3:**
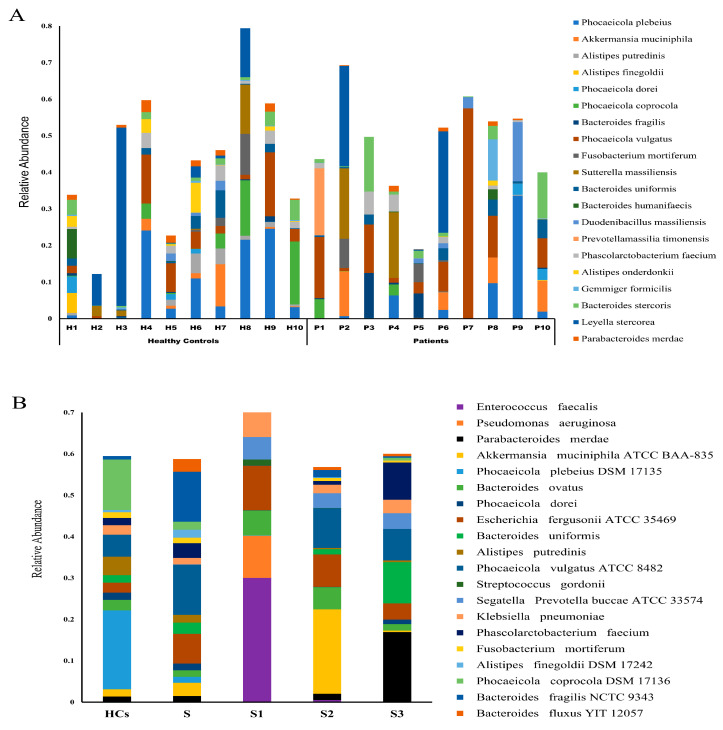
The abundance (**A**) of top thirty bacteria species among healthy controls and patient samples at baseline (S); (**B**) of top twenty bacteria species from healthy controls (HCs), and patient samples at S, S1, S2, and S3.

Further taxonomic profiling revealed significant differences in microbial composition and relative abundance across all study groups. The 20 most abundant bacterial species identified in patient and control cohorts are shown in Figure 3A. Species-level analyses indicated that *E. faecalis* and *Akkermansia muciniphila* as the most dominant species in S1 and S2 samples, respectively. In contrast, *Bacteroides uniformis*, *Parabacteroides merdae*, and *Phascolarctobacterium faecium* were more abundant in S3 samples (Figure 3B). These species may serve as potential biomarkers for monitoring post-surgical gut microbiota and guiding microbiota-targeted interventions.

Further analysis of the patient gut microbiota before and after surgical resection at species level revealed that sixty-one species differed significantly between pre- and post-operation samples, with S1 harboring the highest number of unique species. Additionally, analysis of the top 20 species among patient samples indicated that S1 samples exhibited the highest relative species abundance among all patient groups (Figure S1). Notably, *E. faecalis* was highly enriched in S1 samples, but nearly absent in S samples. This enrichment showed a progressive decline in S2 and S3 samples. This temporal pattern implicates *E. faecalis* as a potential contributor to post-surgical complications, underscoring the need for targeted microbial interventions to enhance patient recovery and treatment outcomes.

### 3.4. Shifts in Opportunistic Gut Microbiota Following CRC Surgery

**Figure 4:**
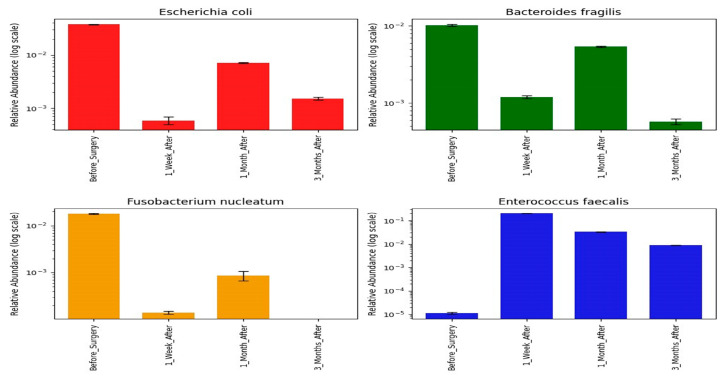
The relative abundance of the four targeted opportunistic species in patient samples at S, S1, S2, and S3 based on 16S rRNA gene sequencing analysis.

Given the established role of opportunistic gut bacteria in CRC pathogenesis [42,43], we further analyzed our metagenomics results to evaluate at the species-level, the shifts in the abundance of such bacteria following surgical resection. We focused on four CRC-associated opportunistic species: *E. coli*, *F. nucleatum*, *B. fragilis*, and *E. faecalis*; across all patient samples (Figure 4). Results indicated that *E. coli*, *F. nucleatum*, and *B. fragilis* exhibited the highest relative abundances in S samples, suggesting their potential involvement in CRC pathogenesis. Following surgery, these species demonstrated a significant reduction in relative abundance in S1 samples (*p* < 0.05). A minor rebound was observed in S2, followed by a subsequent decline in S3. This downward trend post-resection suggests a potential therapeutic benefit of surgical resection on reducing microbial drivers of CRC. In contrast, *E. faecalis* was nearly undetectable in S samples but showed a significant increase in S1 (*p* < 0.05), followed by a gradual decline in S2 and S3. This unique post-surgical surge implies a possible role for *E. faecalis* in early postoperative complications, underscoring the need for targeted interventions during this critical period.

To validate these 16S rRNA-based trends, we performed qPCR on *E. coli*, *B. fragilis*, and *E. faecalis* in all patient samples using species-specific primers (Table S1). PCR screening revealed that *B. fragilis* was detected in 20% of patient samples (2/10), *E. coli* in all samples (10/10), while *E. faecalis* in 50% (5/10). Notably, *E. faecalis* was detected in 90% of S1 samples (9/10), but at lower frequencies in other timepoints: 5/10 in S, 6/10 in S2, and 5/10 in S3. Five patients exhibited continuous detection of *E. faecalis* across all timepoints and were therefore selected for longitudinal qPCR-based quantification. Similarly, for *E. coli* and *B. fragilis*, only those patients with consistent detection from S to S3 were included in the qPCR subset.

**Figure 5:**
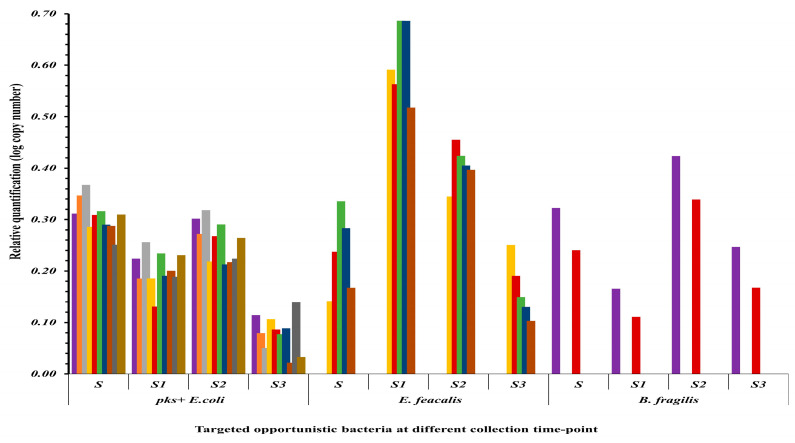
The relative quantification of the three identified opportunistic species in patient samples at S, S1, S2, and S3 based on real-time qPCR.

**Figure 6:**
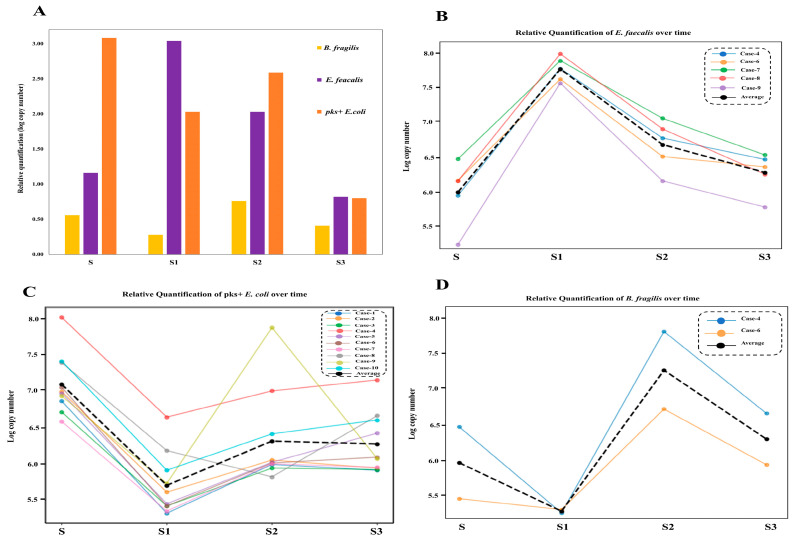
Relative quantities of the three identified opportunistic species in patient samples (**A**) generally and (**B**–**D**) in individual patients at S, S1, S2, and S3 based on real-time qPCR.

The qPCR results analysis showed variations in the gene copy numbers used as proxies for the abundance of the three targeted species over time, with statistically significant reductions in S3 compared to S (*p* < 0.05; Figure 5). Specifically, relative quantification of the *clbB* gene showed significant elevation levels of *pks^+^ E. coli* in S samples than in other patient samples. However, the elevation levels were followed with a steady decline in the levels *clbB* gene in after surgery follow-up (S1, S2 and S3) (Figure 6A). On other hand, the *efa* gene levels for *E. faecalis* peaked in S1 samples (*p* < 0.05), and this was followed by a steady decline through S2 and S3.

Although *B. fragilis* is a recognized CRC-associated pathogen [10,44], it was comparatively less abundant across our study cohort. In samples where it was detected (2/10), the levels of *bft* gene for this bacterial species were lower in S samples than those of *E. coli*. However, there was a slight increase in *B. fragilis* levels in S2 samples compared to S and S1; and this was followed by a decline at S3. The case-by-case qPCR analysis revealed a generally consistent pattern of declining opportunistic bacterial abundance post-surgery, particularly by the S3 time point, with the exception of *E. faecalis* in S1 (Figure 6B–D). Additionally, a clinical outlier patient was observed in which a transient spike in *pks^+^ E. coli* abundance at S2 was displayed, followed by a sharp drop at S3 (Figure 6C).

### 3.5. Functional and Metabolic Analysis of the Gut Microbiota

Functional profiling of the gut microbiota, inferred using PICRUSt2, revealed distinct metabolic and biosynthetic pathway patterns among the study cohort. Principal Component Analysis (PCA) of KEGG orthologous pathways demonstrated a clear divergence along PC1 axis (85% variance) of S1 samples from healthy controls and all other patient groups (Figure S2A), indicating a markedly different functional profile at this collection time point. This divergence was further supported by Statistical Analysis of Metagenomic Profiles (STAMP), which showed a more isolated distribution of S1 samples, compared to a relatively overlapping clustering between S, S2, S3 and healthy controls (Figure S2B).

**Figure 7:**
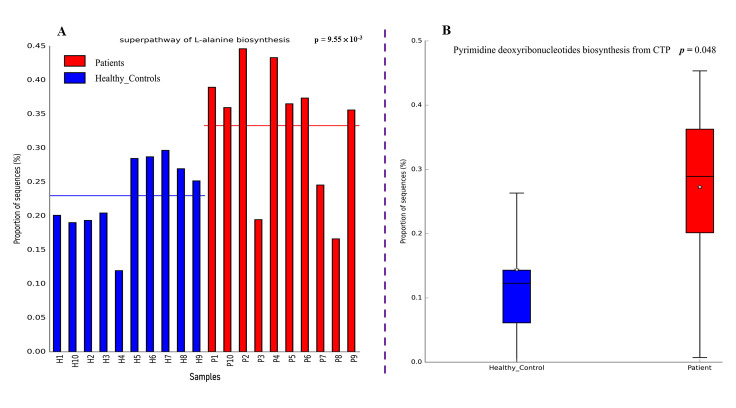
Bar and box plots showing significantly enriched bacterial metabolic pathways: (**A**) energy metabolism-related pathway enrichment in patients; (**B**) microbial adaptive response pathway enrichment in patients.

Nevertheless, most energy metabolism-related and pro-tumorigenic microbial metabolic functional pathways were enriched in patients as indicated in (Figure 7A). Specifically, several functional pathways including chorismate biosynthesis from 3-dehydroquinate, NAD biosynthesis I, pyruvate fermentation to acetate and lactate II, UMP biosynthesis, and glycolysis III were significantly enriched (*p* < 0.05) in patient samples than in healthy controls (Figure S3). Additionally, patients exhibited significantly higher levels of pyruvate deoxyribonucleotide biosynthesis from cytidine triphosphate (CTP) (*p* = 0.048) (Figure 7B). This may reflect a potential microbial adaptive response to host inflammation, altered nutrient availability, or post-surgical physiological stress in the CRC environment.

## 4. Discussion

Although genetic and epigenetic factors have long been established as critical contributors to CRC development [45,46], an expanding body of evidence underscores the role of gut microbiota dysbiosis in CRC pathogenesis [47,48]. Surgical resection remains the primary curative treatment [49,50]; however, postoperative complications, particularly those of bacterial origin, such as postoperative ileus, surgical site infections, and anastomotic leakage, remain clinically burdensome [51,52]. These complications often arise despite optimal surgical techniques and aseptic protocols [51], which highlights the urgent need for preventative strategies against such bacteria. One of such strategies would involve understanding the alterations in gut microbiota composition following surgery, to identify such species potentially associated with complications. Amounting evidence suggests that these microbial alterations contribute to postoperative outcomes [25,53]. This implies that understanding such dynamics is crucial, particularly in guiding interventions such as the targeted re-introduction of beneficial commensals via probiotics therapy [54,55].

While previous studies have broadly profiled the gut microbial composition after CRC surgery, they offer a generalized compositional overview [39,56]. Given the significant role played by pro-carcinogenic bacteria in the pathogenesis and recurrence of CRC [57,58], our pilot study presents a focused investigation into the postsurgical dynamics of such bacteria in CRC patients.

Despite not showing statistically significant differences in microbial community between patients and healthy controls, there was a higher number of unique microbial species shared between healthy controls and patients (Figure 1A). This observation was initially explained by the composition of our study cohort, where healthy controls were close relatives of patients, typically residing in the same household or local environment. However, our results were consistent with recent studies conducted in unrelated populations, which also reported minimal differences in gut microbiota diversity between patients and healthy controls [39,59]. Family members often share environmental exposures and dietary habits, which can lead to partially overlapping microbial communities. This overlap may explain the higher number of unique microbial species shared between healthy controls and patients. However, evidence from twin cohorts and large-scale microbiome genome-wide association studies shows that heritable factors such as host genetics contribute only modestly to gut microbial variability. For instance, a recent twin study reported that genetic factors accounted for just 8.8% of microbial composition [60]. This indicates that microbial variation among people may not only be attributed to differences in familial or environmental proximity, but also other confounders such as host immune response [61,62,63]. Recent studies show that gut immune signaling during infection can significantly shape microbial ecology [64]. As a result, innate immune mechanisms such as antimicrobial peptide production, maintenance of the mucosal barrier, and pattern-recognition receptor pathways, selectively restrict or permit colonization by specific taxa, thereby supporting beneficial microbes while suppressing harmful ones [62]. Adaptive immunity further refines these interactions through secretory IgA (sIgA) coating, which influences microbial adhesion and persistence [65]. Given that CRC activates both innate and adaptive immune responses [66], these immune-mediated pressures likely play an important role in reshaping the gut microbial community in CRC patients, leading to the reported differences in gut microbiota diversity between patients and healthy controls.

Within the patient groups, the alpha diversity in S1 was significantly different from that in S, S2 and S3. Additionally, beta diversity analysis further indicated how the bacterial community structure in S1 was distinct from the other patient samples. These findings likely reflect the effects of pre-operative and post-operative procedures such bowel preparation and prophylactic antibiotics administration to gut microbiota [26,67]. Such procedures are known to perturb the gut microbial ecosystem, weakening colonization resistance and increasing susceptibility to opportunistic pathogens [56]. This may thus explain the increased abundance of pathogenic bacteria such as *E. faecalis* and *Pseudomonas aeruginosa* in S1 (Figure S1**)** which are known for surgical site infections and post-surgery complications [68,69].

Furthermore, we observed a significant increase in the relative abundance of the beneficial bacterium *Akkermansia muciniphila* in S2 samples (*p* = 0.0006) compared to other patient group samples (Figure 3B). Emerging evidence supports the role of *A. muciniphila* in enhancing gut barrier integrity, particularly through upregulation of tight junction proteins such as Claudin-5 and Claudin-8, which are critical in sealing the intestinal epithelium and preventing paracellular leakage [70,71]. Additionally, *A. muciniphila* has been shown to mitigate peritonitis and promote colonic wound healing via a MyD88-dependent pathway of the innate immunity [72]. Therefore, increased abundance of this species during the recovery phase (S2) may suggest its potential association with favorable post-surgical outcomes [73,74]. These findings raise the possibility that *A. muciniphila* may serve as a prognostic biomarker of intestinal recovery and mucosal restoration following colorectal surgery. Additionally, it can be harnessed as a candidate for next-generation probiotic development to accelerate mucosal repair [75].

Furthermore, significant increase in the relative abundance we observed in the core members of the commensal gut microbiota such as *B. uniformis*, *P. merdae*, and *P. faecium* in S3 samples, [76,77,78], and their observed depletion in our study following colorectal resection are consistent with previous studies [28,79,80]. The re-emergence of these species in S3 suggests progressive gut microbial restoration following perturbations and may serve as a potential biomarker panel for monitoring gut microbial recovery following colorectal surgery.

We further elucidated the microbial dynamics in CRC by examining the relative abundance of opportunistic gut microbiota species previously implicated in CRC pathogenesis [57]. Initially, we focused on the 16S rRNA gene sequencing analysis of *E. coli*, *F. nucleatum*, *B. fragilis*, and *E. faecalis* across all patient sampling time points. Notably, *E. coli*, *F. nucleatum*, and *B. fragilis* were significantly more abundant in S samples compared to subsequent follow-ups. This pattern is consistent with prior studies linking these species to CRC progression [81,82]. However, the relative abundancies of each of these species reduced significantly in S1 samples, suggesting a possible disruption of the microbial niche, probably caused by bowel preparation and post-surgery medical care [37]. There was a slight increase in their relative abundance in S2 samples which decreased again later in S3 samples. This may imply that such opportunistic bacteria thrive in the cancerous ecological niche and when disrupted through resection, it affects their proliferation and eventually their abundance. This trend supports our hypothesis that surgical resection may transiently or permanently disrupt the ecological niche of such opportunistic bacteria thus removing them or reducing their abundance in the patient gut.

Conversely, the abundance of E. faecalis increased significantly in S1 samples, coinciding with the period immediately following surgery. This species has been implicated in post-operative complications, particularly anastomotic leaks [20,83]. Mechanistically, *E. faecalis* is known to produce collagenolytic enzymes and activate matrix metalloproteinase-9 (MMP-9) [68,84], which degrade extracellular matrix components critical for surgical wound integrity. Furthermore, *E. faecalis* readily forms biofilms at surgical sites [85], enhancing its resistance to standard perioperative antibiotic prophylaxis and facilitating persistent infection.

The elevated abundance of *E. faecalis* in S1 is consistent with previous reports in CRC and other gastrointestinal surgeries, reinforcing its potential role in adverse surgical outcomes [20,86,87]. The gradual decline of *E. faecalis* in S2 and S3 may suggest recovery from complications, though there was no statistical significance (*p* = 0.93) between the reported complications and the decline of this species, probably due to sample size. Taken together, these findings highlight *E. faecalis* as a candidate prognostic biomarker for post-surgical complications and underscore the need for targeted antimicrobial or microbiome-modulating strategies to mitigate its clinical impact.

We validated the 16S rRNA sequencing findings by performing qPCR analysis, targeting three key opportunistic pathogens: *E. faecalis*, *E. coli*, and *B. fragilis*. The PCR screening for the *clbB* indicated a potential high prevalence of pks⁺ *E. coli*, in our cohort. Future studies should aim to explore the extent of this prevalence among CRC patients in our population for potential targeted therapeutic strategies. The qPCR analysis revealed that the highest relative quantification of *pks⁺* *E. coli* was in S samples, with a progressive decline from S1 to S3. This pattern mirrored our 16S rRNA analysis results, aligning with our hypothesis that surgical resection disrupts colonization and re-colonization of CRC-associated bacteria. Future studies should delve into factors that may lead to re-colonization of *pks⁺* *E. coli* among surgically treated CRC patients.

In line with sequencing results, *E. faecalis* exhibited a marked increase in relative abundance in S1 samples across all five patients in whom it was detected. For *B. fragilis*, qPCR revealed a modest but non-significant increase in relative abundance during S2. This may potentially reflect the species’ ability to utilize host-derived substrates such as mucin and N-linked glycans during the recolonization phase [88,89]. These glycoproteins are typically upregulated following epithelial injury and are involved in tissue repair and mucosal barrier restoration [90,91]. A recent study revealed that *B. fragilis* efficiently metabolize these complex glycans [88], which may transiently support its proliferation during S2 follow-up. However, by S3, a decrease in its relative abundance was observed, suggesting microbial rebalancing and suppression of opportunistic expansion as the gut microbiota recovers [53,92]. This aligns with our hypothesis that the abundance of opportunistic bacteria is decreased post-operation.

Overall, the qPCR quantification trends were consistent across time points for all the three pathogens. One exception was a single patient in which *pks⁺* *E. coli* abundance deviated from the trend in S2 (Figure 6C). Notably, no significant correlation was found between *pks⁺* *E. coli* abundance and the reported surgical site infection in this case, suggesting that other cofounders may have contributed to this deviation. Given the established roles of these opportunistic taxa in CRC pathogenesis, the observed post-operative decline in their abundance may hold clinical significance, particularly in light of concerns that their persistence may contribute to disease recurrence and chemoresistance [17,33,34].

The analysis of predicted bacterial metabolic and functional pathways revealed a significant clustering between S1 and other samples in our study cohort. This distinctive shift in the functional profile is likely driven by pre-operative bowel preparations and post-operative infection control measures affecting the gut microbiota [56,67]. However, there was a gradual stabilization in gut microbiota observed by a somewhat relatable clustering of functional pathways in S2 and S3 to that of healthy controls. This implies that the major disturbances in the gut microbiota caused by surgery [25,93] may gradually be stabilized with time. Future studies should observe this gradual stability for a longer period to establish whether a complete return to a normal gut microbiota is achieved.

Furthermore, we observed a significant increase in the metabolic pathways that support bacterial growth such as Chorismate biosynthesis, NAD biosynthesis, Pyruvate fermentation to acetate and lactate, among others in CRC patients compared to healthy controls (Figure S3). This could be reflecting a pro-tumorigenic microbial metabolic environment created by opportunistic microbiota in the patient gut [94]. Additionally, significant elevation of pathways such as pyrimidine nucleotide biosynthesis in patients could be driven by the rapid turnover and proliferation of specific opportunistic bacterial species associated with tumor growth and inflammation [95].

Our study was not without limitations. As a pilot study conducted at a single center, the aforementioned small sample size could have affected our statistical power to detect associations with clinical or demographic variables. Additionally, all the recruited CRC patients were diagnosed with rectal cancer. As colon and rectal cancers have different molecular biology and possibly inhabited by different microbial species, our results may not be generalized for all CRCs [96]. In addition, healthy control samples were collected at baseline with no subsequent follow-up sampling to match the CRC patient sample groups. Although matching all CRC samples with healthy control samples at all sampling timepoints would provide a better comparison, our study did not focus on prevalence of opportunistic bacteria among healthy control samples. More to this, dietary patterns were not strictly monitored particularly for CRC patients given that changes in diet can influence gut microbial composition [97,98]. As this was a pilot study, future studies should put into account the aforementioned limitations for a more comprehensive and generalizable study.

Nevertheless, the present study provided valuable insight into the dynamic changes in the abundance of opportunistic gut microbiota after surgical resection in CRC patients. Further validation studies should be carried out in a multi-center and a larger study cohort, with longer follow-up duration to examine the possibility of complete restoration of the gut microbiota after resection.

## 5. Conclusions

This study demonstrates that colorectal resection reduces the abundance of opportunistic gut microbiota composition in CRC patients. Species such as *E. coli* were enriched in patients prior to surgery, however their relative abundance declined with time in post-surgery. Additionally, *E. faecalis* was highly abundant in S1 follow-up, which signifies its potential as a prognostic biomarker for post-operative complications. In contrast, the abundance of beneficial taxa including *A. muciniphila*, *B. uniformis*, *P. merdae*, and *P. faecium* increased in later follow-ups of S2 and S3. This highlights the potential of these species as indicators of positive surgery outcomes. Our study findings provide positive insights amidst concerns that persistent opportunistic bacteria may contribute to disease recurrence and chemoresistance. Future validation studies with longer follow-up durations should provide more comprehensive understanding of the fate of opportunistic bacteria and the potential for complete gut microbiota restoration post-surgery.

## Data Availability

The original contributions presented in this study are included in the article/Supplementary Materials. Further inquiries can be directed to the corresponding author.

## Supplementary Materials

The following supporting information can be downloaded at: https://www.mdpi.com/article/10.3390/microorganisms13122818/s1, Table S1: Primer sequences used for real-time qPCR targeting three opportunistic gut microbiota. Table S2: Demographic overview of the study population. Figure S1: The abundance of top twenty bacteria species from patient samples at S, S1, S2, and S3. Figure S2: Functional and metabolic profiles of the gut microbiota between CRC patients and healthy controls. (A) Principal Coordinate analysis (PCoA) of PICRUSt2-projected functional profiles between healthy controls and CRC patients at S, S1, S2, and S3 (*p* < 0.05, ANOVA Tukey–Kramer test). (B) The host hoc plot showing significantly enriched functional pathways in patient and healthy control samples. It presents mean percentage proportional difference and the significant scores (*p* < 0.05) of enriched pathways. (C) Significantly enriched pathways between patient and health controls. Figure S3: Differential enrichment of bacterial growth–associated metabolic pathways in colorectal cancer (CRC) patients compared to healthy controls. Pathways shown include chorismate biosynthesis, NAD biosynthesis I, pyruvate fermentation to acetate and lactate II, UMP biosynthesis, and glycolysis III. Relative pathway abundances were analyzed using STAMP (v2.1.3) and statistical significance was assessed by two-sided Welch’s *t*-test with Benjamini–Hochberg correction. References [10,99,100] are cited in the supplementary materials.
