# Supplementary material for: Deciphering the Post-Operative Dynamics of Opportunistic Gut Microbiota in Colorectal Cancer Patients
Source: Microorganisms. 2025 Dec 11;13(12):2818. doi: 10.3390/microorganisms13122818 (PMC12735874; doi:10.3390/microorganisms13122818)
Supplement: Supplementary file 1 [file microorganisms-13-02818-s001.zip › microorganisms-4007422-supplementary.pdf]

Table S1: Primer sequences used for real-time qPCR targeting three opportunistic gut microbiota

Target Bacteria	Target Gene	F & R primers (5'–3')	PCR Product (bp)	qPCR Conditions	Reference
Bacteroides fragilis (ETBF)	*bft-1*	**R**: CGGAATCATTATGCTATCGGGTA<br>**F**: CGAGGGGCATCAGGAAGAA	136bp (36 cycles)	94° C for 5 min<br>94 °C for 45 secs<br>52 °C for 30 secs<br>72°C for 45 sec<br>72° C for 5 min	[1]
Escherichia coli (pks-positive)	*clbB*	**F**: GCGCATCCTCAAGAGTAAATA<br>**R**: GCGCTCTATGCTCATCAACC	280 bp (35 cycles)	94 °C for 5 min<br>94 °C for 30 secs<br>60 °C for 45 secs<br>72 °C for 30 secs<br>72 °C for 2 min	[2]
Enterococcus faecalis	*E. faecalis 16S rRNA*	**F**: ATCAAGTACAGTTAGTCTTTATTAG<br>**R**: ACGATTCAAAGCTAACTGAATCAGT	113-bp (40 cycles)	95°C for 2 min<br>95°C for 15 secs<br>55°C for 10 secs<br>72°C for 30 secs<br>72 °C for 5 min	[3]

Table S2: Demographic overview of the study population.

Variable	Healthy Controls	CRC Patients
**Number**	15	10
**Age, year, mean (IQR)**	51.3 (26.0)	63.0 (8.5)
**Sex (Female/Male), n**	8\7	5\5
**BMI, mean (IQR)**	23.6 (6.0)	23.8 (3.0)
**Tumor Location**	NA	Rectum

Figure S1

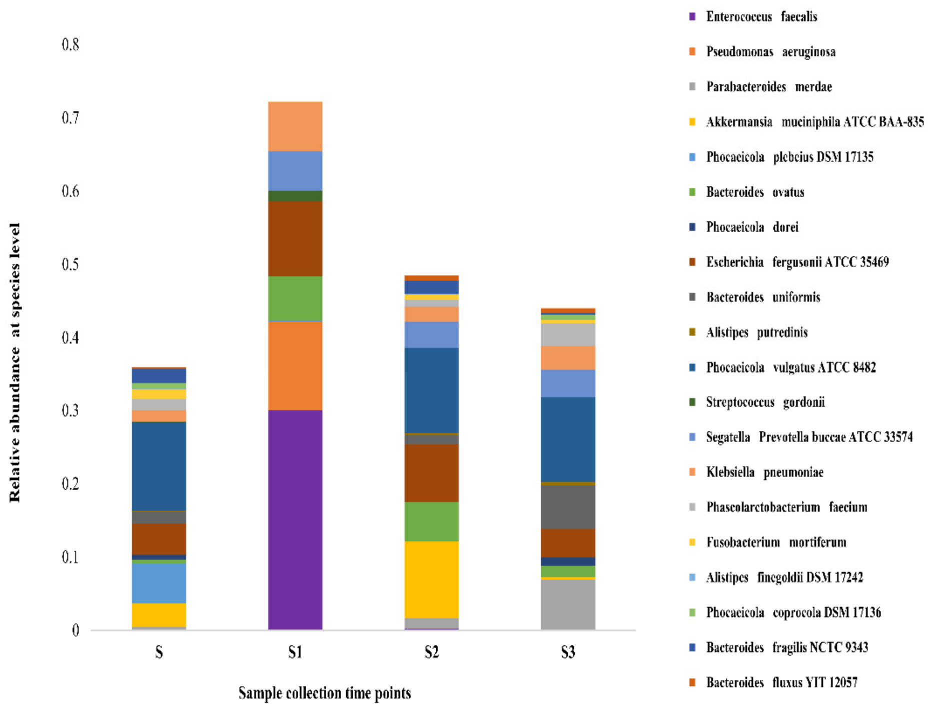

Figure S1: The abundance of top twenty bacteria species from patient samples at S, S1, S2, and S3

Figure S2

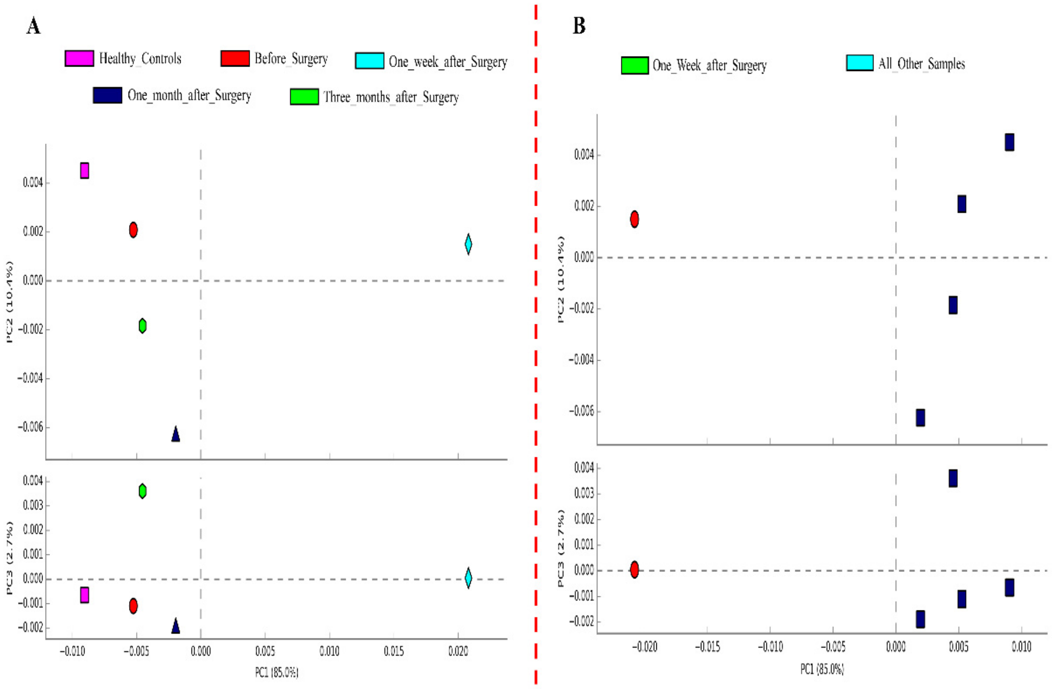

Figure S2: Functional and metabolic profiles of the gut microbiota between CRC patients and healthy controls. (A) Principal Coordinate analysis (PCoA) of PICRUSt2-projected functional profiles between healthy controls and CRC patients at S, S1, S2, and S3 ($p < 0.05$, ANOVA Tukey-Kramer test). (B) The host hoc plot showing significantly enriched functional pathways in patient and healthy control samples. It presents mean percentage proportional difference and the significant scores ($p < 0.05$) of enriched pathways. (C) Significantly enriched pathways between patient and health controls

Figure S3

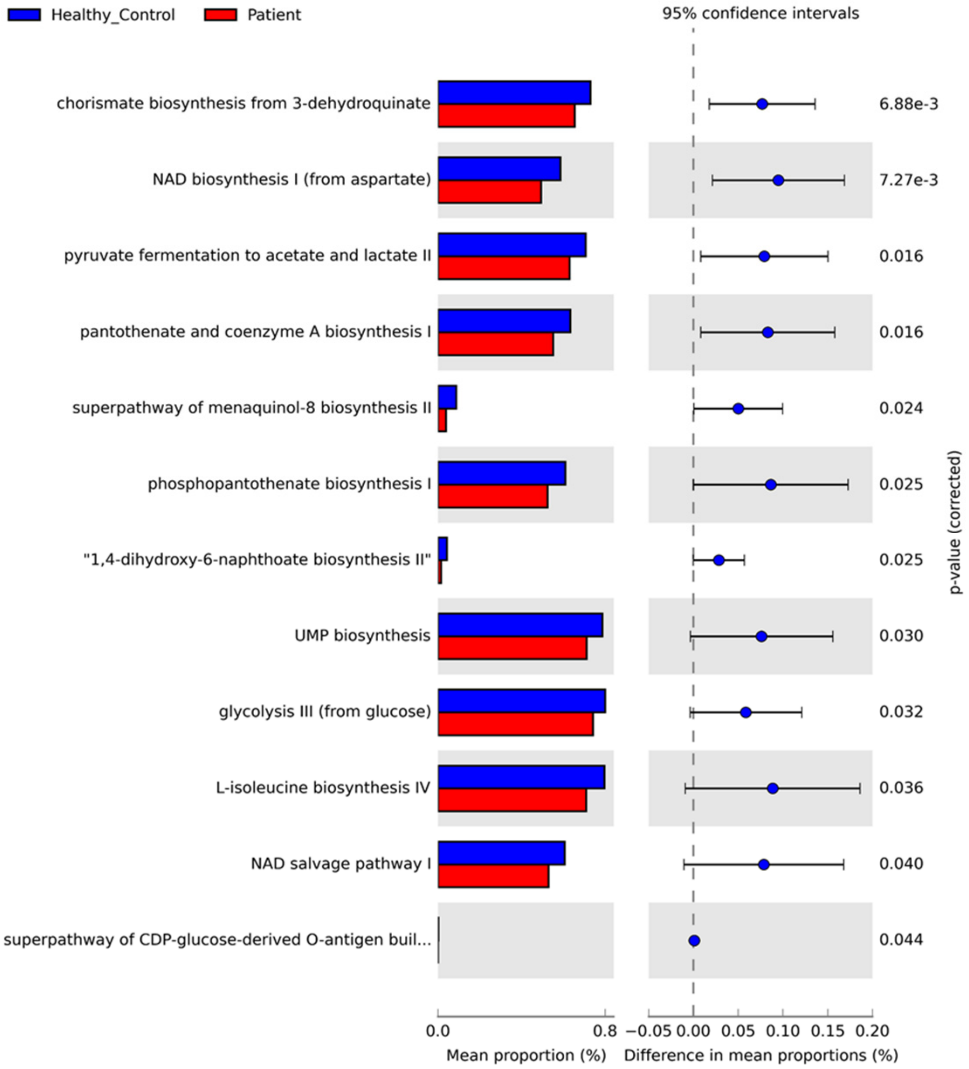

Figure S3: Differential enrichment of bacterial growth–associated metabolic pathways in colorectal cancer (CRC) patients compared to healthy controls. Pathways shown include chorismate biosynthesis, NAD biosynthesis I, pyruvate fermentation to acetate and lactate II, UMP biosynthesis, and glycolysis III. Relative pathway abundances were analyzed using STAMP (v2.1.3) and statistical significance was assessed by two-sided Welch's t-test with Benjamini–Hochberg correction.

References

1. Dadgar-Zankbar, L.; Shariati, A.; Bostanghadiri, N.; Elahi, Z.; Mirkalantari, S.; Razavi, S.; Kamali, F.; Darban-Sarokhalil, D. Evaluation of enterotoxigenic Bacteroides fragilis correlation with the expression of cellular signaling pathway genes in Iranian patients with colorectal cancer. *Infectious agents and cancer* **2023**, *18*, 48.
2. Iyadorai, T.; Mariappan, V.; Vellasamy, K.M.; Wanyiri, J.W.; Roslani, A.C.; Lee, G.K.; Sears, C.; Vadivelu, J. Prevalence and association of pks+ Escherichia coli with colorectal cancer in patients at the University Malaya Medical Centre, Malaysia. *PloS one* **2020**, *15*, e0228217.
3. Geravand, M.; Fallah, P.; Yaghoobi, M.H.; Soleimanifar, F.; Farid, M.; Zinatizadeh, N.; Yaslianifard, S. Investigation of enterococcus faecalis population in patients with polyp and colorectal cancer in comparison of healthy individuals. *Arquivos de Gastroenterologia* **2019**, *56*, 141-145.
